# Coupled microbiome analyses highlights relative functional roles of bacteria in a bivalve hatchery

**DOI:** 10.1186/s40793-021-00376-z

**Published:** 2021-03-31

**Authors:** Emma Timmins-Schiffman, Samuel J. White, Rhonda Elliott Thompson, Brent Vadopalas, Benoit Eudeline, Brook L. Nunn, Steven B. Roberts

**Affiliations:** 1 Department of Genome Sciences, University of Washington, 3720 15th Ave NE, Seattle, WA 98195 USA; 2 School of Aquatic and Fishery Sciences, University of Washington, 1122 Boat St., Seattle, WA 98195 USA; 3 Taylor Shellfish Hatchery, 701 Broadspit Rd., Quilcene, WA 98376 USA; 4 Mason County Public Health, 415 N 6th St., Shelton, WA 98584 USA; 5 Washington Sea Grant, University of Washington, 3716 Brooklyn Ave NE, Seattle, WA 98105 USA

**Keywords:** Microbiome, Shellfish, Geoduck, Larvae, Metagenomics, Metaproteomics

## Abstract

**Background:**

Microbial communities are ubiquitous throughout ecosystems and are commensal with hosts across taxonomic boundaries. Environmental and species-specific microbiomes are instrumental in maintaining ecosystem and host health, respectively. The introduction of pathogenic microbes that shift microbiome community structure can lead to illness and death. Understanding the dynamics of microbiomes across a diversity of environments and hosts will help us to better understand which taxa forecast survival and which forecast mortality events.

**Results:**

We characterized the bacterial community microbiome in the water of a commercial shellfish hatchery in Washington state, USA, where the hatchery has been plagued by recurring and unexplained larval mortality events. By applying the complementary methods of metagenomics and metaproteomics we were able to more fully characterize the bacterial taxa in the hatchery at high (pH 8.2) and low (pH 7.1) pH that were metabolically active versus present but not contributing metabolically. There were shifts in the taxonomy and functional profile of the microbiome between pH and over time. Based on detected metagenomic reads and metaproteomic peptide spectral matches, some taxa were more metabolically active than expected based on presence alone (*Deltaproteobacteria*, *Alphaproteobacteria*) and some were less metabolically active than expected (e.g., *Betaproteobacteria*, *Cytophagia*). There was little correlation between potential and realized metabolic function based on Gene Ontology analysis of detected genes and peptides.

**Conclusion:**

The complementary methods of metagenomics and metaproteomics contribute to a more full characterization of bacterial taxa that are potentially active versus truly metabolically active and thus impact water quality and inter-trophic relationships.

**Supplementary Information:**

The online version contains supplementary material available at 10.1186/s40793-021-00376-z.

## Background

Microbial communities are known to be closely associated with aquatic species and to be important regulators and/or indicators of macrofaunal health (e.g. [1]). Shifts in marine microbiomes can forecast impending disease or death in bivalves and can thus be important biomarkers for ecosystem health (e.g., [2]). The same dynamics between host and microbiome occur in commercial aquaculture, where microbiome dynamics may hold the key to understanding previously unexplained massive larval mortality events.

Aquatic systems are home to dynamic interactions among microbial species, macrofauna, and the physical environment, the outcomes of which determine ecosystem health. Understanding which taxa are present in a given system is a first step towards uncovering these dynamics, which can lead to more accurate predictions and modeling of ecosystems. However, especially for microbes, detected presence does not accurately predict metabolic contribution to the ecosystem [3–5]. Microbiome taxonomy is poorly correlated with environmental variables, whereas metabolic potential of functional groups of microbes is well predicted by environment [6]. Many microbes will produce different metabolites, dependent upon local biotic and abiotic conditions, and thus interact differently with the ecosystem [7–9]. Herein lies a limitation of DNA markers in characterizing an active microbiome. Establishing the presence of a particular microbial taxon and then extrapolating its ecosystem function could easily misrepresent the true impact of the microbial community on the system. By combining DNA (metagenomics) with protein abundance (metaproteomics) we can achieve a substantially more accurate understanding of microbial metabolic contributions in a given environment.

Metaproteomics datasets from aquatic systems are establishing a foundation for better understanding the roles that microbes play as they respond to different environmental conditions. As oceans rapidly change in response to increasing pollution and emissions, bacterial communities will respond functionally to shifts in pH [10] and temperature [11], among other changes. Environmentally-driven physiological shifts in microbiome function could lead to changes in nutrient cycling and biogeochemical cycles. If there is functional redundancy across taxa in a microbiome [6] then some metabolic activities could be maintained. This type of community compensation and balance would be difficult to decipher in a metagenomics dataset alone. Understanding microbial community function is essential to fully characterizing the dynamic interactions in an ecosystem context.

Here, we characterize the microbiome of a shellfish hatchery at two different pH during the culture of Pacific geoduck clam larvae. We combine metagenomics and metaproteomics to better understand how microbial presence overlaps with or differs from microbial metabolic activity. The datasets support important foundational knowledge for better understanding microbial dynamics during shellfish aquaculture.

## Methods

### Larval rearing and microbiome sampling

Water samples were taken from a commercial hatchery for microbiome analysis on days 1, 5, 8 and 12 of a grow-out trial where Pacific geoduck larvae (4 days post fertilization on day 1) were held at two pH levels (Additional file 1). Larval growth was suppressed at low pH, following a typical trend of bivalve larval response to low pH that has been described elsewhere (e.g., [12]). Water was pumped into a commercial shellfish hatchery from ~ 100 ft. deep in Dabob Bay, WA and maintained at pH 7.1 or 8.2 to mimic the low pH detected in Hood Canal, WA (contingent to Dabob Bay) and open ocean pH, respectively. The pH 8.2 treatment water was buffered with sodium bicarbonate, and CO_2_ aeration was used to maintain water at pH 7.1. It should be noted that there could be secondary influences from the sodium bicarbonate treatment and CO_2_ aeration, however, from here on out we will refer to differences in terms of pH. Each pH treatment had its own header tank for treating water where pH was maintained; flow rate into the header tanks was 1.8 gallons per minute (gpm) and into each treatment tank was 0.6 gpm. Three replicate 200 L tanks (flow-through) were used for each pH treatment. Larvae were fed a mix of flagellates and diatoms (*Tisochrysis lutea, Nannochloropsis oculata, Chaetoceros muelleri, Pavlova lutheri, Rhodomonas salina, Tetraselmis suecica*) with 30,000 cells/mL of algae in the larval tanks at day 2 post-fertilization, 40,000 cells/mL by day 3, and 50,000 cells/mL by day 4 through the end of the experiment. Water (3.8 L) was collected from larval tank effluent filtered through serial Swinnex filters (Sigma-Aldrich, Saint Louis, MO) of 5, 0.7, and 0.22 μm. The 0.22 μm filters were folded and frozen in individual plastic bags at − 80 °C.

### Metagenomics

DNA for library construction was isolated from 0.22 μm filters from a single tank from each pH treatment from days 5, 8, and 12 for pH 8.2 and days 1, 8, and 12 for pH 7.1. DNA was isolated with the DNeasy Blood and Tissue Kit (Qiagen, Hilden, Germany), following a modified version of the manufacturer’s Gram-Positive Bacteria protocol. Briefly, filters were unfolded and placed in plastic tubes (1.7 mL) and incubated with Buffer Al (400 μl) and proteinase K (50 μL) overnight at 56 °C. The next morning, ethanol (100%, 400 μl) was added to each sample and samples were eluted in Buffer AE (50 μl). Samples were quantified on a Qubit 3.0 with the Qubit 1x dsDNA HS Assay Kit (Invitrogen, Carlsbad, CA) using 5 μl of sample.

Libraries were prepared for multiplexing using a Nextera DNA Flex Kit (Illumina, San Diego, CA) following the manufacturer’s protocol for DNA quantities < 10 ng, with the following adjustments. Polymerase chain reaction (PCR) steps for library preparation were performed in thin-walled PCR tubes (200 μl) with a PTC-200 thermalcylcer (MJ Research, Bio-Rad Laboratories, Hercules, CA); magnetic separations were performed in tubes (1.7 mL) using a DynaMag2 (Invitrogen). Libraries were quantified on a Qubit 3.0 with the Qubit 1x dsDNA HS Assay Kit (Invitrogen). Library quality was assessed on a 2100 Bioanalyzer with a High Sensitivity DNA Kit (Agilent, Santa Clara, CA). The average fragment size was ~ 530 bp. The six metagenome libraries were pooled and sequenced on an Illumina HiSeqX System with a 150 bp paired-end read length.

Sequencing reads were quality trimmed using TrimGalore (0.4.5 [13];). Reads were subjected to two rounds of trimming; initial quality and adapter trimming, followed by removing 20 bp from the 5′ end of each read. Pre- and post-trimming reads were assessed using FastQC (0.11.7 [14];) and MulitQC (1.6.dev0 [15];).

Gene prediction with the trimmed sequencing reads was performed using MetaGeneMark (v3.38 [16];) to produce a translated metagenome to create a database for metaproteomic protein inference.

Trimmed sequencing reads were functionally annotated with a combination of MEGAN6 (v 6.18.3 [17];) and DIAMOND BLASTx (v 0.9.29 [18];). Annotation with DIAMOND BLASTx [18] was run against NCBI nr database (downloaded September 25, 2019). The resulting DAA files were processed for importing into MEGAN6 with the add-meganizer tool using the following MEGAN6 mapping files: prot_acc2tax-Jul2019X1.abin, acc2interpro-Jul2019X.abin, acc2eggnog-Jul2019X.abin. This work was facilitated through the use of advanced computational, storage, and networking infrastructure provided by the Hyak supercomputer system at the University of Washington.

To perform taxonomic classification, meganized DAA files were imported and converted to RMA6 files via the MEGAN6 graphical user interface “Import from BLAST” dialog, using the default Naive LCA settings and the following mapping files: prot_acc2tax-Jul2019X1.abin, acc2interpro-Jul2019X.abin, acc2eggnog-Jul2019X.abin, acc2seed-May2015xx.abin. All mapping files were downloaded from the MEGAN6 website (https://software-ab.informatik.uni-tuebingen.de/download/megan6/old.html).

Diversity index (Shannon-Weaver and Simpson-Reciprocal) values were determined using MEGAN6 by generating a Comparison with the Normalized Reads setting.

### Metaproteomics

Filters (0.22 μm) from two separate tanks at each pH treatment and all four time points (days 1, 5, 8, 12) (*n* = 8) were opened in a cell culture dish on ice. Four washes of ice cold 50 mM NH_4_HCO_3_ (1 mL) were used to rinse the cells from the filters. The wash containing cells was centrifuged at 10,0000 rpm for 30 min. Liquid was removed from the tubes, leaving pelleted cells. Pellets were resuspended in 50 μm NH_4_HCO_3_ with 6 M urea (100 μl). Each sample was sonicated three times (Sonic Dismembrator Model 100, Fisher Scientific; power set between 1 and 2) and chilled between sonications. Protein digestion and peptide desalting followed the protocol outlined in [19].

Metaproteomics data were acquired on a Q-Exactive mass spectrometer (ThermoFisher, Waltham, MA). Both the pre-column (3.5 cm) and analytical column (30 cm) were packed in-house with Dr. Maisch (Ammerbuch, Germany) C18 3 μm beads. Peptide spectra were collected from 5 μl injections of 1 μg total peptides in triplicate over a 60 min gradient of 5–35% acetonitrile. Samples were run in randomized groups of four with a blank injection in between each group and a quality control standard (Pierce Peptide Retention Time Calibration mix + bovine serum albumin, ThermoFisher) after every second group of four. Proteomics data can be found in the ProteomExchange PRIDE repository under the accession number PXD020692.

Raw mass spectrometry files were searched against a database that included the translated metagenome (see above) and common lab contaminants (cRAPome.org) with Comet 2018.01 rev.2 [21, 22]. After Comet, the TransProteomic Pipeline (TPP) was run to statistically score confident peptide spectra using Peptide prophet and infer proteins with Protein Prophet [23, 24]. Results from the TPP were analyzed with Abacus with an FDR cut-off of 0.01 (combined file ProteinProphet probability of 0.93) to generate consensus peptide and protein assignments across the entire experimental dataset (Additional file 2 [25]). Only proteins with at least 2 unique peptides identified across all experimental replicates were included in downstream analyses. Technical replicates were averaged for the analysis. Normalized spectral abundance (NSAF) values, calculated by Abacus, were used to analyze metaproteomic data across time and pH treatments with non-metric multidimensional scaling plot (NMDS) based on a Bray-Curtis dissimilarity matrix and analysis of similarity (ANOSIM) with the vegan package [26] in R v 3.6.3.

The change in detected taxa and Gene Ontology (GO) terms (i.e. abundance of peptides associated with a specific taxonomic group or GO term) were plotted in R using the peptide spectral matches (PSMs) or reads for a specific taxa/GO term normalized to all PSMs or reads for a mass spec run/library. The first time point in the plots was set to 0 and for a subsequent time point (n) were [ratio of reads or PSMs]_n_-[ratio of reads or PSMs]_n-1_. The ratios of PSMs versus the ratios of reads were plotted on an x-y plot with a linear model line of best fit and corresponding R^2^ value to determine how well reads and PSMs corresponded at each day and pH.

For comparison of statistically significant functional and taxonomic differences between pH treatments at each time point, metaGOmics [27] was used to compare proteins inferred between pH and between days within each pH treatment. All Comet search results (see above) were analyzed together with percolator [28] and the resulting file was parsed into individual mass spectrometry experiment files. Peptide spectral matches for each peptide were averaged within a day and pH treatment so that a single results file for each day/pH was uploaded to metaGOmics. The background proteome for metaGOmics was the metagenome-derived proteins inferred in the Comet-to-Percolator pipeline. Only prokaryotic sequences were used for the analysis since a preliminary analysis of the data revealed confounding contamination from bivalve and algal peptides. The portal for the metaGOmics analysis can be found here: https://www.yeastrc.org/metagomics/viewUploadedFasta.do?uid=QCqX3ZlvD4KkDZ2B. Taxonomic and GO results were considered significant if the metaGOmics Laplace corrected q-value was less than or equal to 0.05.

## Results

### Metagenomics

**Fig. 1:**
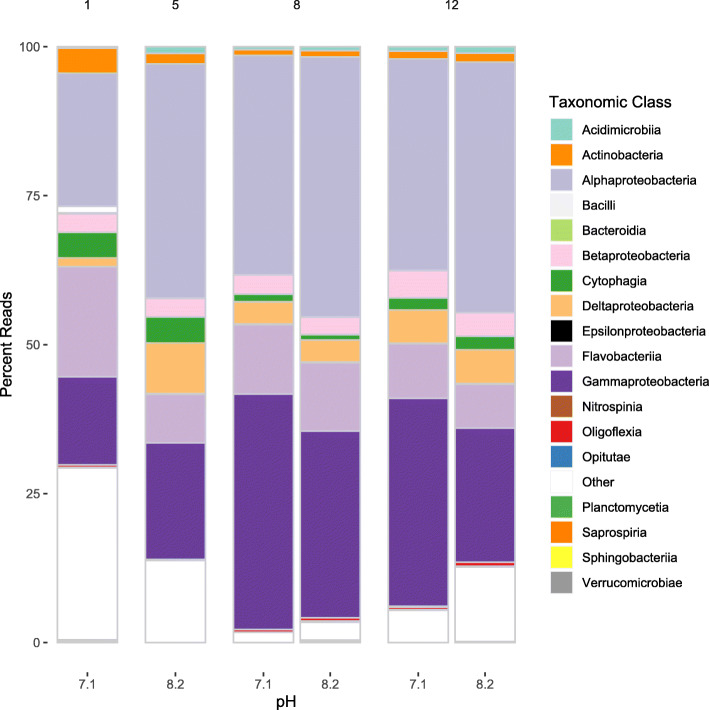
Abundance of taxonomic groups based on the metagenomic dataset for each day and pH. Time points (days) are shown at the top of the plot, while pH is at the bottom. There were no metagenomic libraries sequenced for day 1, pH 8.2 or day 5, pH 7.1

DNA sequencing yielded a total 489 million paired sequencing reads across the six libraries. Raw sequencing data is available in the NCBI sequencing read archive BioProject PRJNA649049. Quality trimming resulted in 474.1 million paired reads that were used for annotation and taxonomic assignment. Sequences were assigned to 26 distinct taxonomic Classes (Additional file 3) and to 62 distinct Gene Ontology terms (Additional file 4). The Shannon-Weaver index (H) was stable across time and pH at a value of about 5, as was the Simpson’s reciprocal index (1/D) (Additional file 5). Through time, the largest changes in Class abundance was observed in *Gammaproteobacteria*, and *Alphaproteobacteria* (Fig. 1).

### Metaproteomics

**Fig. 2:**
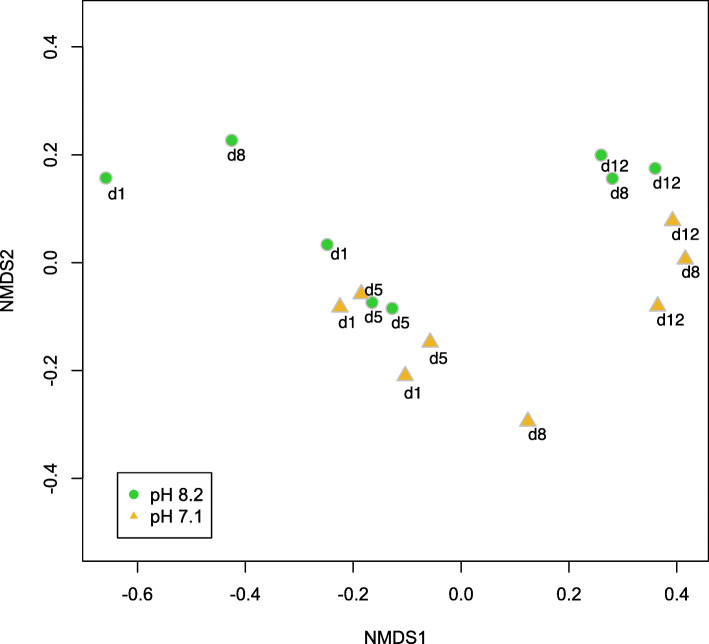
Non-metric multidimensional scaling (NMDS) plot of 983 proteins in the metaproteomic dataset. Metaproteomes from low pH (7.1) communities are represented by yellow triangles and those from high pH (8.2) communities are represented by green circles. Timepoints for days 1 through 12 are indicated with annotations “d1”, “d5”, “d8”, and “d12”

**Fig. 3:**
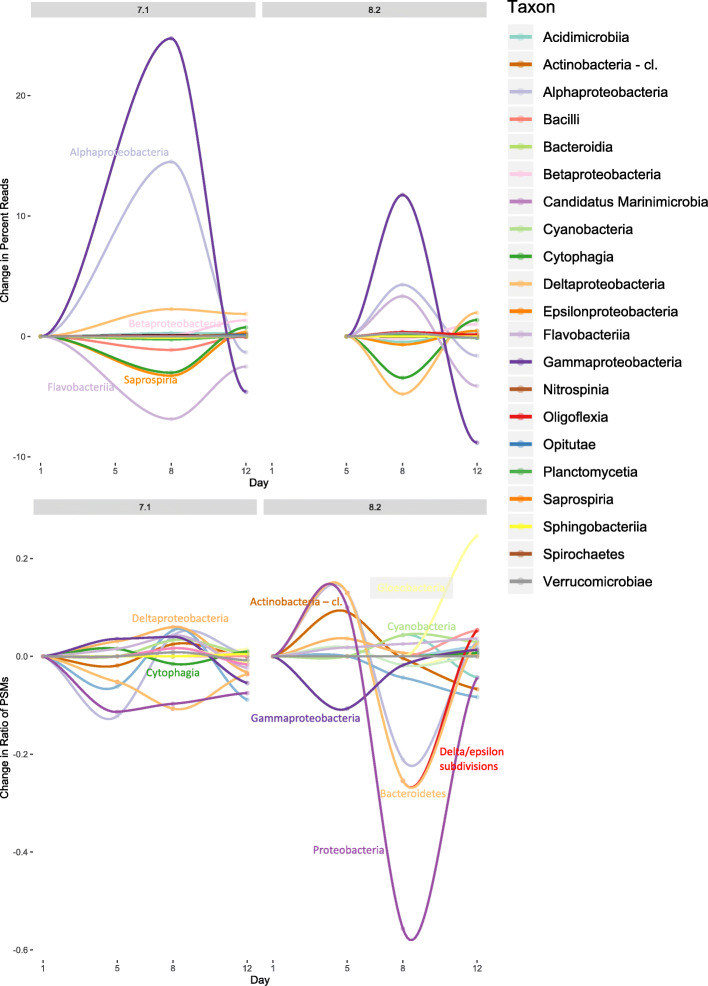
Relative changes in the abundances of metagenomic reads and metaproteomic peptide spectral matches (PSMs) over the 12 day experiment at the two different pH. Each line represents a unique taxonomic group

Across all samples, 983 proteins with at least 2 unique peptides across all replicates were inferred from the mass spectrometry data (Additional file 6). There was a difference in bacterial community protein abundance over time (Day 1 through 12) (ANOSIM *R* = 0.4123, *p* = 0.001), but not by pH (*R* = 0.04129, *p* = 0.207) (Fig. 2). Generally, metaproteomes clustered into early (Days 1 and 5) and late (Days 8 and 12) time points across axis 1 of the NMDS (Fig. 2). Proteins that are strongly, positively loaded along axis 1 of the NMDS are the driving force behind the proteome differentiation. There were six proteins with axis 1 loadings of at least 0.8 and a *p*-value < 0.001: elongation factor 1a, ammonia monooxygenase beta subunit, uncharacterized symporter, putative biopolymer transport protein ExbB homolog, oligopeptide binding protein AppA, and an unannotated protein. The metabolically active fraction of the hatchery water microbiome (i.e. the taxa inferred from the metaproteomics dataset) represented 25 taxonomic groups from Kingdom through Class levels (Fig. 3).

### Taxonomy

The prokaryotic taxonomic community in hatchery water varied by time and pH, and patterns of variability differed between the metagenomic and metaproteomic datasets (Fig. 3). R^2^ values for the correlation between metagenomic reads and metaproteomic peptide spectral matches deviated from 1, revealing a disconnect between taxonomic presence (detected genes) and taxonomic activity (abundance of proteins), although the correlation between metagenomic and metaproteomic abundances was high for days 8 and 12 at pH 7.1 (*R*^2^ ~ 0.7) (Additional file 7). Across both pH, *Deltaproteobacteria* and *Alphaproteobacteria* had higher metabolic activity than would be expected from abundance based on metagenomic reads, whereas *Betaproteobacteria*, *Cytophagia*, *Gammaproteobacteria*, and *Flavobacteriia* had lower activity. A subset of the taxa detected in the metagenomics dataset were not metabolically active based on our metaproteomics technique (Additional file 8).

### Function

**Fig. 4:**
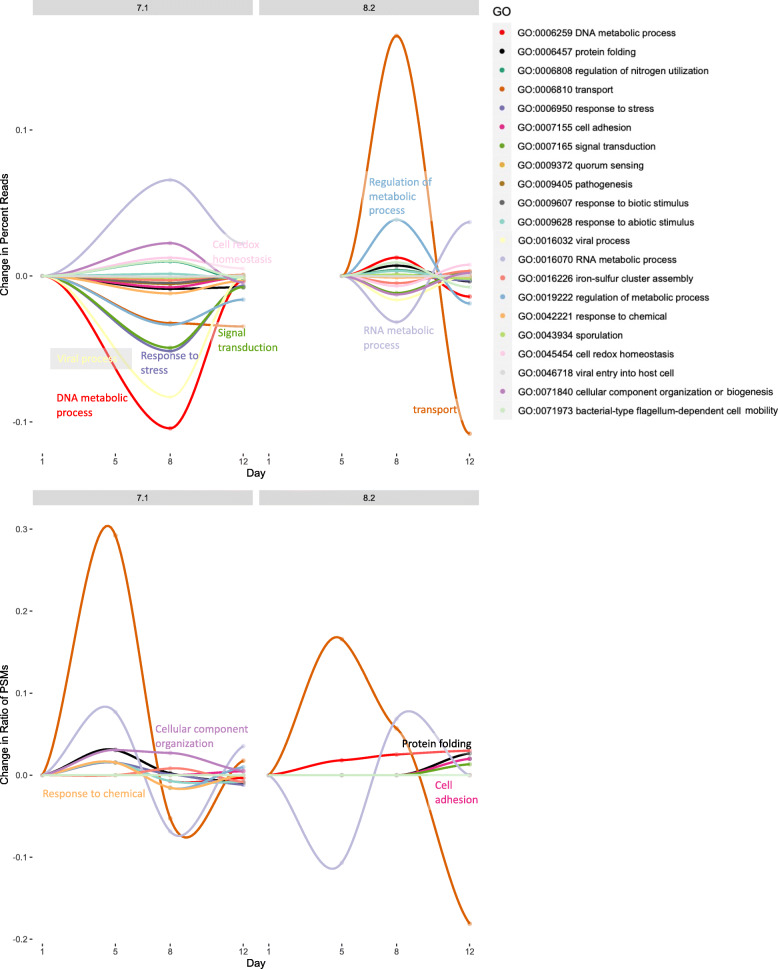
Relative changes in the abundances of metagenomic reads and metaproteomic peptide spectral matches (PSMs) over the 12 day experiment at the two different pH. Each line represents a unique Gene Ontology term

The relative abundance of metagenomic reads for protein functional categories compared to metaproteomic peptide spectral matches for these same functional categories varied across time and pH (Fig. 4). The differences suggest a mismatch between potential and realized physiological function of the hatchery microbiome. For example, the PSMs corresponding to the GO term “transport” increased between days 1 and 5 and then decreased on day 8 in pH 7.1, whereas metagenomic reads for “transport” maintained a continuous gradual decrease from day 1 to 12. In general, the changing abundance of PSMs for functional categories was more dynamic than the actual gene abundances. Metaproteomics data for functional categories correlated with the metagenomics data better than for taxonomic groups with R^2^ values ranging from 0.68 to 0.79 (except for pH 7.1, day 1 for which all presented GO terms were not detected in the metaproteomics dataset) (Additional file 9).

### Significant changes over time and pH

**Fig. 5:**
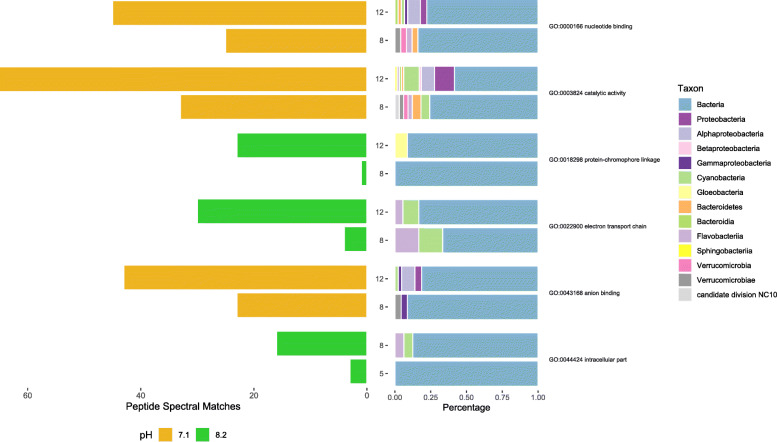
MetaGOmics summary figure of GO terms that were significantly different between time points. The lefthand bars show the total PSMs for each GO term (listed on far right) that was significant in an inter-day comparison for microbial communities at pH 7.1 (yellow) or pH 8.2 (green). The righthand bars show the percent taxonomic contribution to the PSMs for a given day and pH with colors corresponding to taxonomy

MetaGOmics analysis revealed the GO terms that changed significantly over time (Fig. 5; Additional file 10). At pH 8.2 peptides associated with the GO term “intracellular part” increased significantly from day 5 to 8. The contributing taxa to the GO term “intracellular part” were *Bacteria*, *Bacteroidetes*, *Cyanobacteria*, and *Flavobacteriia*. Between days 8 and 12 at pH 8.2 the GO terms “protein-chromophore linkage” and “electron transport chain” saw a significant increase in associated peptides. These peptides were associated with the taxonomic groups *Bacteria*, *Bacteroidetes*, *Cyanobacteria*, *Flavobacteriia*, and *Gloeobacteria*. At pH 7.1, the three GO terms that changed significantly between days 8 and 12 were “nucleotide binding”, “catalytic activity”, and “anion binding”. The taxa that were associated with the peptides in these three GO terms at pH 7.1 were *Bacteria*, *Proteobacteria*, *Cyanobacteria*, *Verrucomicrobia*, *Bacteroidetes*, candidate division NC10, *Betaproteobacteria*, *Alphaproteobacteria*, *Sphingobacteriia*, *Bacteroidia*, *Gammaproteobacteria*, *Flavobacteriia*, *Verrucomicrobia*. There were no GO terms that differed significantly between pH on any of the days.

## Discussion

This is the first study to assess the potential (genomic) versus realized (proteomic) function of a commercial bivalve hatchery microbiome. We characterized the microbial community present in hatchery water over 12 days and at two pH levels during production of Pacific geoduck larvae. ‘Omics tools facilitate high resolution analyses of molecular level processes, but genomics, transcriptomics, proteomics, and metabolomics all have the capability of addressing distinct, occasionally overlapping, sets of hypotheses. Genomics analyses are frequently applied to respond to a diversity of hypotheses in environmental science, because DNA is more stable than RNA and proteins, and genomics, unlike proteomics, does not depend upon a pre-existing database to get results. However, genomics analyses cannot clarify real-time metabolic activity, which limits the interpretation of the data to the potential, rather than realized, function of a gene. In many metagenomics datasets, interpretation extends beyond the limitations of the data to assume realized function. In terms of environmental modeling and predictions, this over-interpretation of data may under- or overestimate community contributions to biogeochemical cycling. The parallel analysis of metagenomics and metaproteomics data herein definitively demonstrates that potential genetic function does not accurately predict which proteins are translated for probable metabolic activity. Metaproteomics analysis provides the additional layer of realized microbial function that allows for fuller interpretation of community biological function. By combining metagenomics and metaproteomics, we have leveraged the increased sensitivity of genomics with the better estimation of physiological activity through metaproteomics.

The relative abundances of bacterial classes in the hatchery microbiome over the entire time period and across both pH was similar between the genomic and proteomic datasets, but variances indicate differences between potential and realized functions of the community. *Alphaproteobacteria* was the most abundant and active bacterial Class, comprising the largest number of metagenomic reads and metaproteomic peptide spectral matches (PSMs). The phylum *Proteobacteria*, which contains the Classes *Alpha-*, *Beta*-, *Delta*-, and *Gammaproteobacteria*, often dominates marine 16SrRNA microbiome datasets associated with hatcheries and shellfish [2, 29–33]. *Gammaproteobacteria*, *Flavobacteriia*, and *Cytophagia* had higher relative abundances in the metagenomic datasets compared to the metaproteomic datasets, suggesting relatively low metabolic activity. *Alphaproteobacteria*, *Gammaproteobacteria*, and *Flavobacteriia* have been found to stabilize microbial ecosystems within arctic mesocosms [34] and may play a similar role in the hatchery microbiome where they dominate in abundance. *Deltaproteobacteria*, *Actinobacteria*, and *Cyanobacteria* were all detected at relatively higher abundances in the metaproteomics dataset than would be expected from abundance of metagenomic reads. These results underline the inherent and potential bias in a single ‘omics dataset.

The largest differences in the hatchery microbiome occurred over time, rather than between pH. Bacterial community diversity in hatcheries is dynamic [29, 30, 35] as the microbiome of incoming water shifts and as the larvae enter different developmental stages. Our microbiome sampling encompassed days 6 through 17 post-fertilization and the larval phase transition from prodissoconch I to II, during which the larvae undergo profound physiological changes. The relative contributions of the different bacterial taxa shifted dramatically over time, as did the potential and realized functions of the community, represented by GO terms. In the metagenomics dataset, *Gammaproteobacteria*, *Alphaproteobacteria*, and *Flavobacteriia* dominated in overall abundance at both pH; however, the dominant taxa for protein abundance were more varied with high counts for *Deltaproteobacteria*, *Alphaproteobacteria*, *Gammaproteobacteria*, *Bacteroidetes*, *Flavobacteriia*, *Cyanobacteria* (including *Gloeobacteria*), and the *Actinobacteria* phylum. There is likely a dynamic relationship between larval shellfish and their surrounding microbiome, which may at least partially dictate which taxa are present and/or active. In an oyster larvae culture, *Alteromondaceae* (*Gammaproteobacteria*) dominated the microbiome during very early larval development, followed by *Flavobacteriaceae* (*Flavobacteriia*) and *Rhodobacteraceae* (*Alphaproteobacteria*) between 1 and 16 days post-fertilization [30]. This larval-microbiome interrelationship may contribute to some of the observed differences in the geoduck larval hatchery microbiome.

Changes in taxonomy do not necessarily reflect shifts in community function, but some physiological pathways did change over time, especially between days 8 and 12. Between days 5 and 8, there was an increase in abundance of peptides associated with the GO term “intracellular part” at pH 8.2 from the taxa *Bacteria*, *Cyanobacteria*, and *Flavobacteriia*. The largest increases in abundance of peptides associated with GO terms occurred between days 8 and 12, with peptides detected associated with “nucleotide binding” (pH 7.1), “catalytic activity” (pH 7.1), “anion binding” (pH 7.1), “protein-chromophore linkage” (pH 8.2), and “electron transport chain” (pH 8.2). These changes suggest an environmental shift that altered community metabolism with peptides in these significantly changing categories contributed by taxa such as *Alphaproteobacteria*, *Betaproteobacteria*, *Gammaproteobacteria*, *Cyanobacteria*, *Sphingobacteriia*, and *Verrucomicrobia*. *Cyanobacteria* may increase photosynthetic activity at increased levels of CO_2_/low pH [36] and it was a significant contributor to changing physiology over time at both pH suggesting a dynamic physiological environmental response. Bacterial communities are sensitive to shifts in the surrounding phytoplankton community since phytoplankton represent an important nutrient source. From mesocosm experiments, bacterial production and enzymatic activity change with the onset and decline of phytoplankton blooms [37, 38]. Since the shellfish hatchery that housed our microbiome relies on water pumped from a natural source, it is possible that the bacterial physiological changes detected are in response to changing environmental conditions in the source water. However, since the microbiome is likely also impacted by its proximity to the rapidly developing larval shellfish, the hypothesis of microbiome-larval interdependence cannot be ruled out. Further work to control for variability in incoming water and larval impacts on the surrounding microbiome would be needed to separate these variables.

Bacterial communities respond to changes in pH mostly through secondary responses to pH impacts on their source of dissolved and particulate organic matter (i.e. phytoplankton) [37–41]. When a measurable bacterial community physiological shift occurs in response to pCO_2_, it may be mediated by availability of nutrients which may, in turn, be controlled by the local primary producers [37, 42]. Primary impacts of pH on bacteria are possible; in a low pH environment, ocean bacteria may physiologically compensate for elevated pCO_2_ (increased H^+^ ions) by up-regulating proton pumping mechanisms to maintain cellular pH homeostasis [11, 43]. Changes in pCO_2_ seem to have little impact on the taxonomic groups detected in a microbiome [34]; there may be, however, some rare taxa that do respond directly to shifts in pCO_2_ [40]. Similar to numerous mesocosm studies, we detected no significant change in peptide abundance between pH treatments, suggesting little physiological impact of pH on the hatchery microbiome. However, it is possible that the live algae that is fed to the larvae and the geoduck larvae themselves were responding to the changes in pH [12, 44], which could have led to secondary impacts on the microbiome. The geoduck larvae were likely stressed at low pH, which can be manifested in metabolic changes and decreases in growth rate [12]. Between the two pH treatments, there were different GO terms that changed significantly over time, perhaps indicating a subtle, secondary impact of pH on the hatchery microbiome. The two GO terms that were significantly differentially abundant between days 8 and 12 at pH 8.2 (“protein-chromophore linkage” and “electron transport chain”) are associated with cellular metabolism. The shifts in peptides associated with these terms suggests a change in metabolic demands or capacity at pH 8.2. The changes at pH 7.1 (“nucleotide binding”, “catalytic activity”, and “anion binding”) also suggest changes to cellular activity, but perhaps through different pathways than at pH 8.2. Bacterial physiology as measured by extracellular enzyme activity and bacterial production sometimes changes in response to pH and/or pH impacts on a nutrient source [37, 38, 42, 43, 45], and a similar physiological impact is likely occurring at the different pH treatments in the hatchery. These results highlight the interconnected nature of aquatic food webs and that the sensitivity of one component can have cascading effects throughout an interconnected system.

## Conclusions

The parallel analysis of metagenomics and metaproteomics datasets provides important insight into the divergence between potential and realized metabolic function of a hatchery microbiome. These data are necessary for establishing a baseline understanding of water quality, which is an essential resource for an economically important industry. There were no significant mortality events among the geoduck larvae that were grown throughout this experiment that co-occurred with our microbiomes. Previous work has found that larval health in commercial hatcheries may be more dependent on the presence of specific microbial taxa that confer immunity, rather than on an influx of harmful microbes [31, 46]. Since bivalve larval immune function biomarkers are detectable from very early in development [47], the timing of the presence of immune-conferring taxa may also be essential for success of a particular cohort. Some of the taxa detected in our study may be part of the microbiome essential for an effective immune response and survival, but it is difficult to discern which taxa this would be without differential mortality between larval cohorts. There is a correlation between host microbiome diversity and the complexity of the immune system, with more stable microbiomes in invertebrates with innate immune systems [48]. It is worth identifying this stable, core microbiome in ecologically and commercially important taxa as a means to better understand mechanisms of environmental response and survival. A dataset has yet to be published that definitively determines why larval cohorts survive or die in a commercial hatchery setting and the current work adds an important piece to solving this costly puzzle.

## Supplementary Information

**Additional file 1.** Size of geoduck larvae over the course of the experiment at pH 8.2 (green) and 7.1 (yellow). Days when ‘omics samples were taken are circled in blue.

**Additional file 2.** Abacus parameters file

**Additional file 3.** Counts of reads in each sequencing library assigned to each taxonomic Class (including reads assigned to descendants of each class).

**Additional file 4.** Counts of reads in each sequencing library assigned to each Gene Ontology term.

**Additional file 5.** Diversity indices for metagenomics data for pH 8.2 (green) and pH 7.1 (yellow). Shannon-Weaver index is represented by the solid lines and Simpson’s Reciprocal index is represented by dashed lines.

**Additional file 6.** Proteins identified in the metaproteomics dataset. Protein names derived from the metagenome are listed in the first column followed by columns of spectral counts for each mass spectrometry run. The last 3 columns contain protein length, a Uniprot annotation resulting from a BLASTp and the corresponding e-value for the BLASTp result.

**Additional file 7.** Plots of the correlation the metagenomic reads and metaproteomic peptide spectral matches for each taxonomic Class identified for each day and pH. R^2^ values are reported on each plot and Classes are labeled.

**Additional file 8.** Representation of tax that are missing from a dataset for a given sampling day or pH. Solid circles indicate that the taxon was present in the metagenomics dataset; an open circle indicates presence in the metaproteomics dataset.

**Additional file 9.** Plots of the correlation the metagenomic reads and metaproteomic peptide spectral matches for each Gene Ontology term identified for each day and pH in the metagenomics dataset. R^2^ values are reported on each plot and colors correspond to Fig. 4.

**Additional file 10.** Results from the MetaGOmics analysis. Each tab contains the results for a different pairwise comparison either between days at a single pH or between pH on a single day.

## Data Availability

The sequenced metagenomics data can be found in NCBI’s Short Read Archive under BioProject PRJNA649049. The metaproteomics dataset can be found in the ProteomeXchange PRIDE repository under accession PXD020692.

## References

[1] King WL, Jenkins C, Seymour JR, Labbate M (2019). Oyster disease in a changing environment: decrypting the link between pathogen, microbiome and environment. Mar Environ Res.

[2] King WL, Jenkins C, Go J, Siboni N, Seymour JR, Labbate M (2019). Characterisation of the pacific oyster microbiome during a summer mortality event. Invertebr Microbiol.

[3] Maier T, Schmidt A, Guell M, Kuhner S, Gavin AC, Aebersold R, Serrano L (2011). Quantification of mRNA and protein and integration with protein turnover in a bacterium. Mol Syst Biol.

[4] Waldbauer JR, Rodrigue S, Coleman ML, Chisholm SW (2012). Transcriptome and proteome dynamics of a light-dark synchronized bacterial cell cycle. PLoS One.

[5] Pavlov MY, Ehrenberg M (2013). Optimal control of gene expression for fast proteome adaptation to environmental change. Proc Natl Acad Sci U S A.

[6] Louca S, Parfrey LW, Doebeli M (2016). Decoupling function and taxonomy in the global ocean microbiome. Science..

[7] Seitzinger SP, Sanders RW, Styles R (2002). Bioavailability of DON from natural and anthropogenic sources to estuarine plankton. Limnol Oceanogr.

[8] Berman T, Bronk DA (2003). Dissolved organic nitrogen: a dynamic participant in aquatic ecosystems. Aquat Microb Ecol.

[9] Amin SA, Parker MS, Armbrust EV (2012). Interactions between diatoms and bacteria. Microbiol Mol Biol Rev.

[10] Bunse C, Lundin D, Karlsson CMG, Akram N, Vila-Costa M, Palovaaraa J, Svensson L, Holmfeldt K, González JM, Calvo E, Pelejero C, Marrasé C, Dopson M, Gasol JM, Pinhassi J (2016). Response of marine bacterioplankton pH homeostasis gene expression to elevated pCO2. Nat Clim Chang.

[11] Burrell TJ, Maas EW, Hulston DA, Law CS (2017). Variable response to warming and ocean acidification by bacterial processes in different plankton communities. Aquat Microb Ecol.

[12] Timmins-Schiffman E, Guzmán JM, Elliott Thompson R, Vadopalas B, Eudeline B, Roberts SB (2020). Dynamic response in the larval geoduck (*Panopea generosa*) proteome to elevated pCO_2_. Ecol Evol.

[13] Krueger F. A wrapper around Cutadapt and FastQC to consistently apply adapter and quality trimming to FastQ files, with extra functionality for RRBS data - FelixKrueger/TrimGalore [Computer software] https://github.com/FelixKrueger/TrimGalore. 2017.

[14] Andrews S. A quality control analysis tool for high throughput sequencing data - s-andrews/FastQC [Computer software] https://github.com/s-andrews/FastQC. 2018.

[15] Ewels P. Aggregate results from bioinformatics analyses across many samples into a single report. - ewels/MultiQC [Computer software] https://github.com/ewels/MultiQC. 2018.

[16] Zhu W, Lomsadze A, Borodovsky M (2010). Ab initio gene identification in metagenomic sequences. Nucleic Acids Res.

[17] Huson DH, Beier S, Flade I, Górska A, El-Hadidi M, Mitra S, Ruscheweyh H-J, Tappu R (2016). MEGAN community edition - interactive exploration and analysis of large-scale microbiome sequencing data. PLoS Comput Biol.

[18] Buchfink B, Xie C, Huson DH (2015). Fast and sensitive protein alignment using DIAMOND. Nat Methods.

[19] Timmins-Schiffman E, May DH, Mikan M, Riffle M, Frazar C, Harvey HR, Noble WS, Nunn BL (2017). Critical decisions in metaproteomics: achieving high confidence protein annotations in a sea of unknowns. ISME J.

[20] Vizcaíno JA, Csordas A, del-Totor N, Dianes JA, Griss J, Lavidas I, Mayer G, Perez-Riverol Y, Reisinger F, Ternent T, Xu QW, Wang R, Hermjakob H (2016). 2016 updates of the PRIDE database and related tools. Nucleic Acids Res.

[21] Eng JK, Jahan TA, Hoopmann MR (2012). Comet: an open source tandem mass spectrometry sequence database search tool. Proteomics..

[22] Eng JK, Jahan TA, Egertson JD, Noble WS, MacCoss MJ (2015). A deeper look into comet - implementation and features. J Am Soc Mass Spectrom.

[23] Keller A, Hesvizhskii AI, Kolker E, Aebersold R (2002). Empirical statistical model to estimate the accuracy of peptide identifications made by MS/MS and database search. Anal Chem.

[24] Nesvizhskii AI, Keller A, Kolker E, Aebersold R (2003). A statistical model for identifying proteins by tandem mass spectrometry. Anal Chem.

[25] Fermin D, Basrur V, Yocum AK, Nesvizhskii AI (2011). Abacus: a computational tool for extracting and pre-processing spectral count data for label-free quantitative proteomic analysis. Proteomics.

[26] Okasanen J, Blanchet FH, Friendly M, Kindt R, Legendre P, McGlinn D, Minchin PR, O’Hara RB, Simpson GL, Solymos P, Stevens MHH, Szoecs E, Wagner H (2019). vegan: community ecology package.

[27] Riffle M, May DH, Timmins-Schiffman E, Mikan MP, Jaschob D, Noble WS, Nunn BL (2018). MetaGOmics: a web-based tool for peptide-centric functional and taxonomic analysis of metaproteomics data. Proteomes..

[28] Käll L, Canterbury JD, Weston J, Noble WS, MacCoss MJ (2007). Semi-supervised learning for peptide identification from shotgun proteomics datasets. Nat Methods.

[29] Stevick RJ, Sohn S, Modak TH, Nelson DR, Rowley DC, Tammi K, Smolowitz R, Lundgren KM, Post AF, Gómez-Chiarri M (2019). Bacterial community dynamics in an oyster hatchery in response to probiotic treatment. Front Microbiol.

[30] Laroche O, Symonds JE, Smith KF, Banks JC, Mae H, Bowman JP, Pochon X (2018). Understanding bacterial communities for informed biosecurity and improved larval survival in Pacific oysters. Aquaculture..

[31] Powell SM, Chapman CC, Bermudes M, Tamplin ML (2013). Dynamics of seawater bacterial communities in a shellfish hatchery. Microbiol Aquat Syst.

[32] Asmani K, Petton B, Le Grand J, Mounier J, Robert R, Nicolas J-L (2016). Establishment of microbiota in larval culture of Pacific oyster, *Crassostrea gigas*. Aquaculture.

[33] Trabal N, Mazón-Suástegui JM, Vázquez-Juárez R, Asenci-Valle F, Morales-Bojórquez E, Romero J (2012). Molecular analysis of bacterial microbiota associated with oyster (*Crassostrea gigas* and *Crassostrea corteziensis*) in different growth phases at two cultivation sites. Microb Ecol.

[34] Wang Y, Zhang R, Zheng Q, Deng Y, Van Nostrand JD, Zhou J, Jiao N (2016). Bacterioplankton community resilience to ocean acidification: evidence from microbial network analysis. ICES J Mar Sci.

[35] Trabal Fernández N, Mazón-Suástegui JM, Vázquez-Juárez R, Asenci-Valle F, Romero J (2014). Changes in the composition and diversity of the bacterial microbiota associated with oysters (*Crassostrea corteziensis, Crassostrea gigas,* and *Crassostrea sikamea*) during commercial production. FEMS Microb Ecol.

[36] O’Brien PA, Morrow KM, Willis BL, Bourne DG (2016). Implications of ocean acidification for marine microorganisms from the free-living to the host-associated. Front Mar Sci.

[37] Grossart H-P, Allgaier M, Passow U, Riebesell U (2006). Testing the effect of CO2 concentration on the dynamics of marine heterotrophic bacterioplankton. Limnol Oceanogr.

[38] Allgaier M, Riebesell U, Vogt M, Thyrhaug R, Grossart H-P (2008). Coupling of heterotrophic bacteria to phytoplankton at different pCO2 levels: a mesocosm study. Biogeosci Discuss.

[39] Liu J, Weinbauer MG, Maier C, Dai M, Gattuso J-P (2010). Effect of ocean acidification on microbial diversity and on microbe-driven biogeochemistry and ecosystem functioning. Aquat Microb Ecol.

[40] Roy A-S, Gibbons SM, Schunck H, Owens S, Caporaso JG, Sperling M, Nissimov JI, Romac S, Bittner L, Muhling M, Riebesell U, LaRoche J, Gilbert JA (2013). Ocean acidification shows negligible impacts on high-latitude bacterial community structure in coastal pelagic mesocosms. Biogeosciences..

[41] Hornick T, Bach LT, Crawfurd KJ, Spilling K, Achterberg EP, Woodhouse JN, Schulz KG, Brussard CPD, Riebesell U, Grossart H-P (2017). Ocean acidification impacts bacteria-phytoplankton coupling at low-nutrient conditions. Biogeosciences..

[42] Sala MM, Aparicio FL, Balagué V, Boras JA, Borrull E, Cardelús C, Cros L, Gomes A, Lópeq-Sanz A, Malits A, Martínez RA, Mestre M, Movilla J, Sarmento H, Vázquez-Domínguez E, Vaqué D, Pinhassi J, Calbet A, Calvo E, Gasol JM, Pelejero C, Marrasé C (2016). Contrasting effects of ocean acidification on the microbial food web under different trophic conditions. ICES J Mar Sci.

[43] Westwood KJ, Thomson PG, van den Enden RL, Maher LE, Wright SW, Davidson AT (2018). Ocean acidification impacts primary and bacterial production in Antarctic coastal waters during austral summer. J Exp Mar Biol Ecol.

[44] Bautista-Chamizo E, Sendra M, De Orte MR, Riba I (2019). Comparative effects of seawater acificiation on microalgae: single and multispecies toxicity tests. Sci Total Environ.

[45] Piontek J, Lunau M, Händel N, Borchard C, Wurst M, Engel A (2009). Acidification increases microbial polysaccharide degradation in the ocean. Biogeosci Discuss.

[46] Karim M, Zhao W, Rowley D, Nelson D, Gomez-Chiarri M (2013). Probiotic strains for shellfish aquaculture: protection of eastern oyster, *Crassostrea virginica*, larvae and juveniles against bacterial challenge. J Shellfish Res.

[47] Tirapé A, Bacque C, Brizard R, Vandenbulcke F, Boulo V (2007). Expression of immune-related genes in the oyster *Crassostrea gigas* during ontogenesis. Dev Comp Immunol.

[48] Woodhams DC, Bletz MC, Becker G, Bender HA, Buitrago-Rosas D, Diebboll H, Huynh R, Kearns PJ, Jueneman J, Kurosawa E, LaBumbard BC, Lyons C, McNally K, Schliep K, Shankar N, Tokash-Peters AG, Vences M, Whetstone R (2020). Host-associated microbiomes are predicted by immune system complexity and climate. Genome Biol.

