# Supplementary figures and images for: Coupled microbiome analyses highlights relative functional roles of bacteria in a bivalve hatchery
Source: Environ Microbiome. 2021 Mar 31;16:7. doi: 10.1186/s40793-021-00376-z (PMC8066469; doi:10.1186/s40793-021-00376-z)

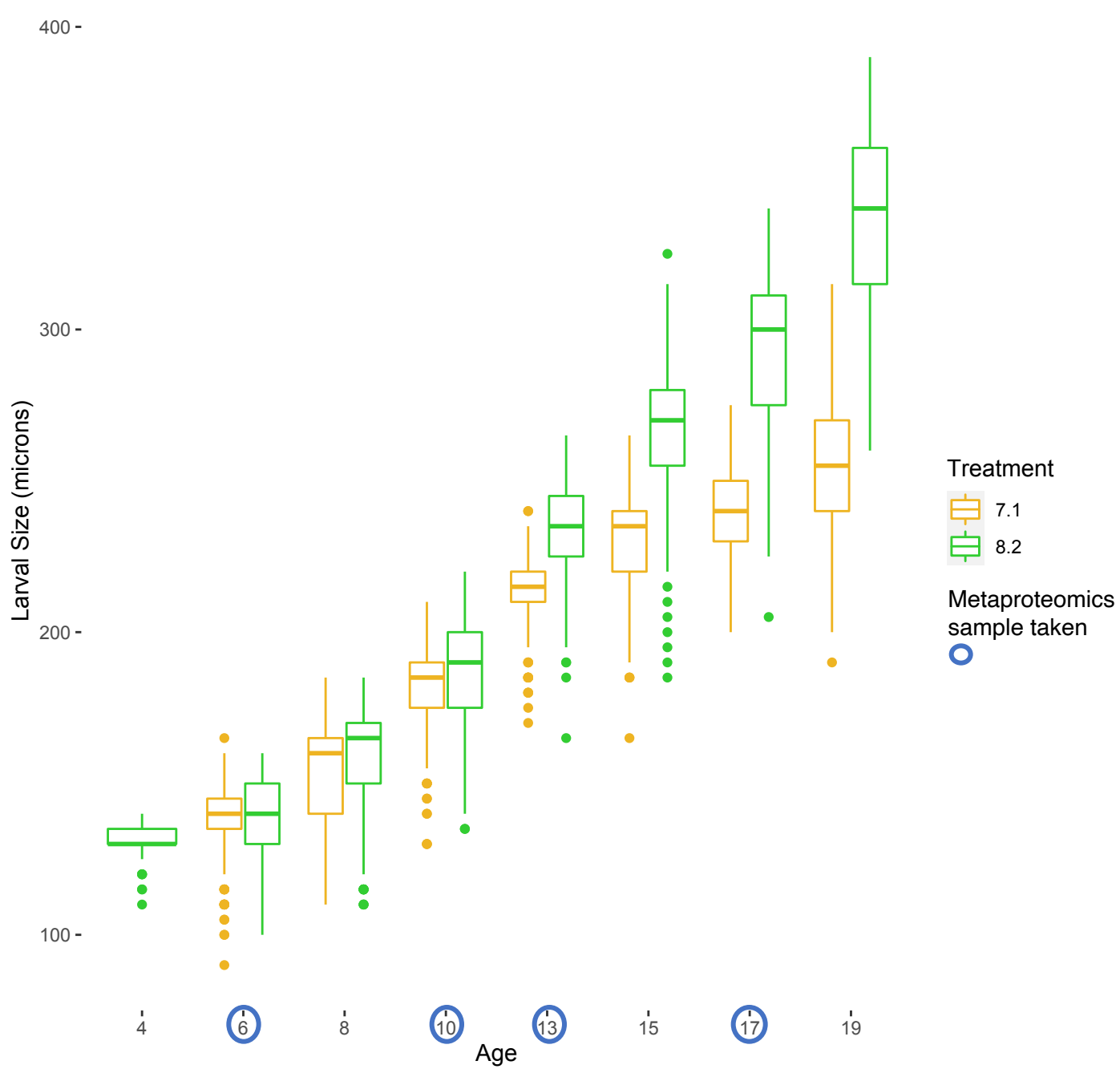

Supplement: Supplementary file 1 — Additional file 1. Size of geoduck larvae over the course of the experiment at pH 8.2 (green) and 7.1 (yellow). Days when ‘omics samples were taken are circled in blue. [file 40793_2021_376_MOESM1_ESM.pdf]

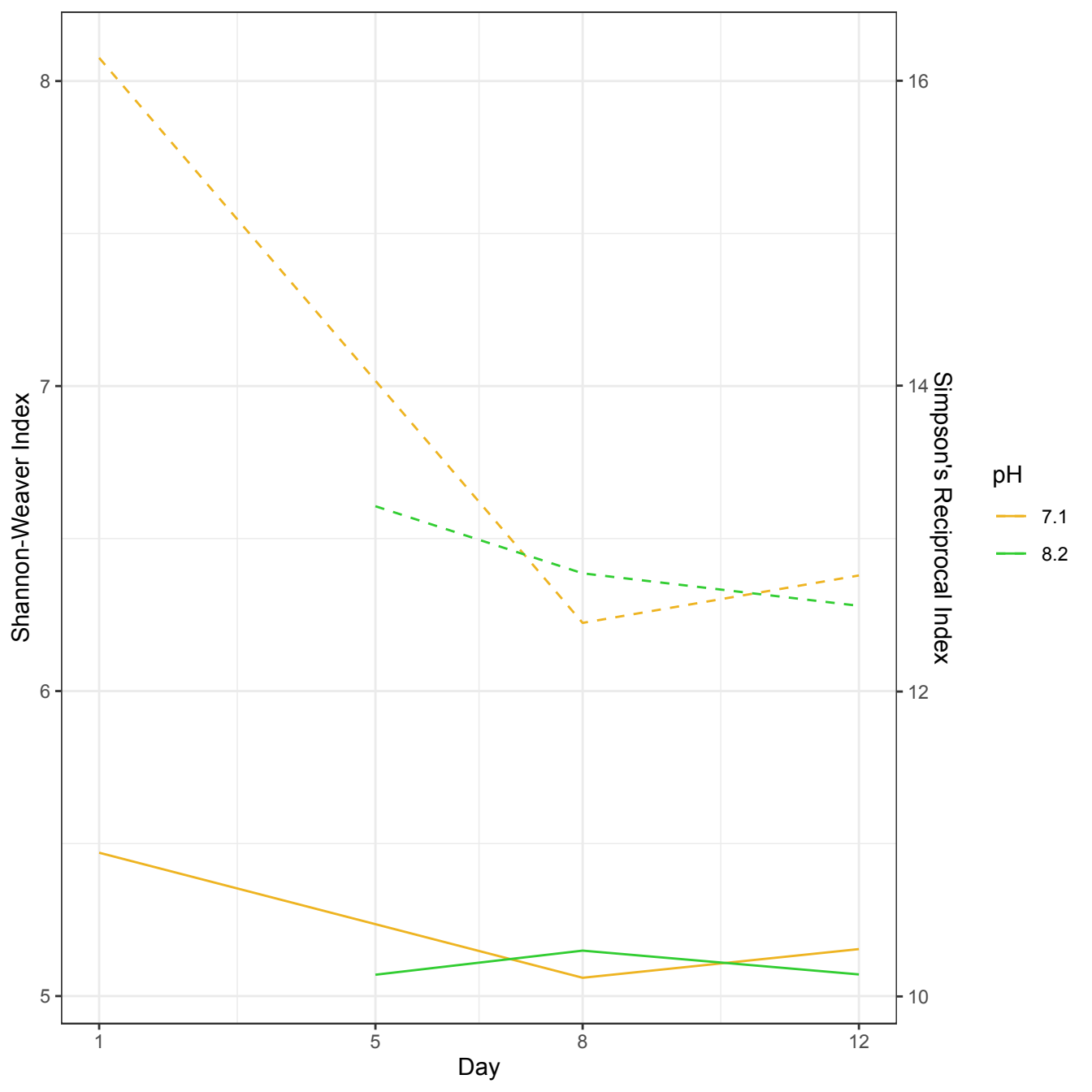

Supplement: Supplementary file 5 — Additional file 5. Diversity indices for metagenomics data for pH 8.2 (green) and pH 7.1 (yellow). Shannon-Weaver index is represented by the solid lines and Simpson’s Reciprocal index is represented by dashed lines. [file 40793_2021_376_MOESM5_ESM.pdf]

Ratio of Metaproteomic PSMs in Run

1

7.1

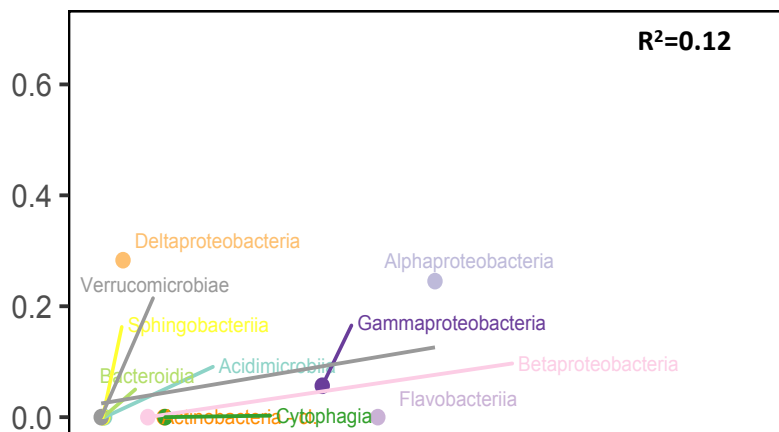

5

8.2

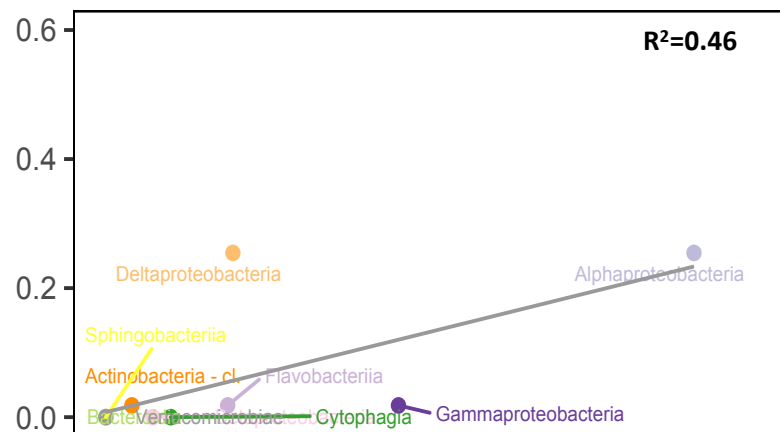

8

7.1

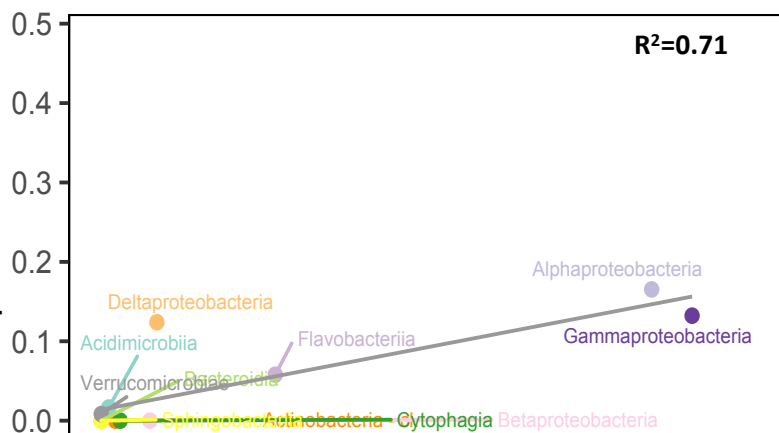

8

8.2

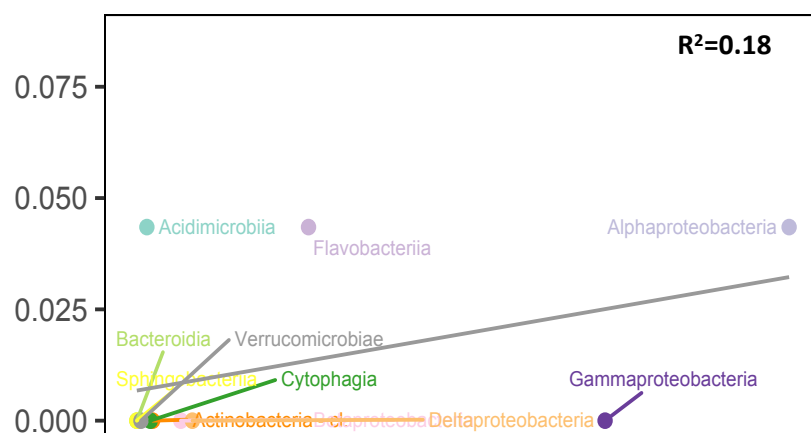

12

7.1

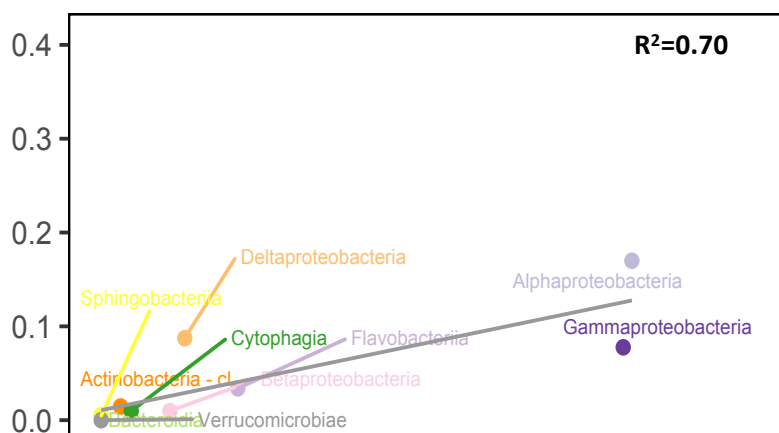

12

8.2

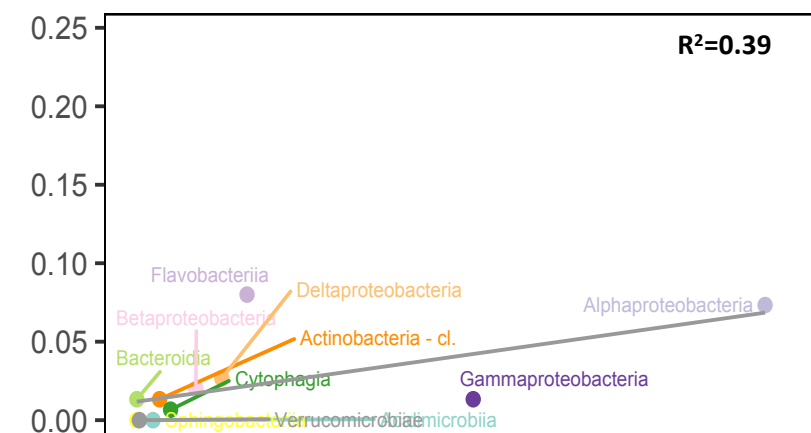

Ratio of Metagenomic Reads in Library

Supplement: Supplementary file 7 — Additional file 7. Plots of the correlation the metagenomic reads and metaproteomic peptide spectral matches for each taxonomic Class identified for each day and pH. R2 values are reported on each plot and Classes are labeled. [file 40793_2021_376_MOESM7_ESM.pdf]

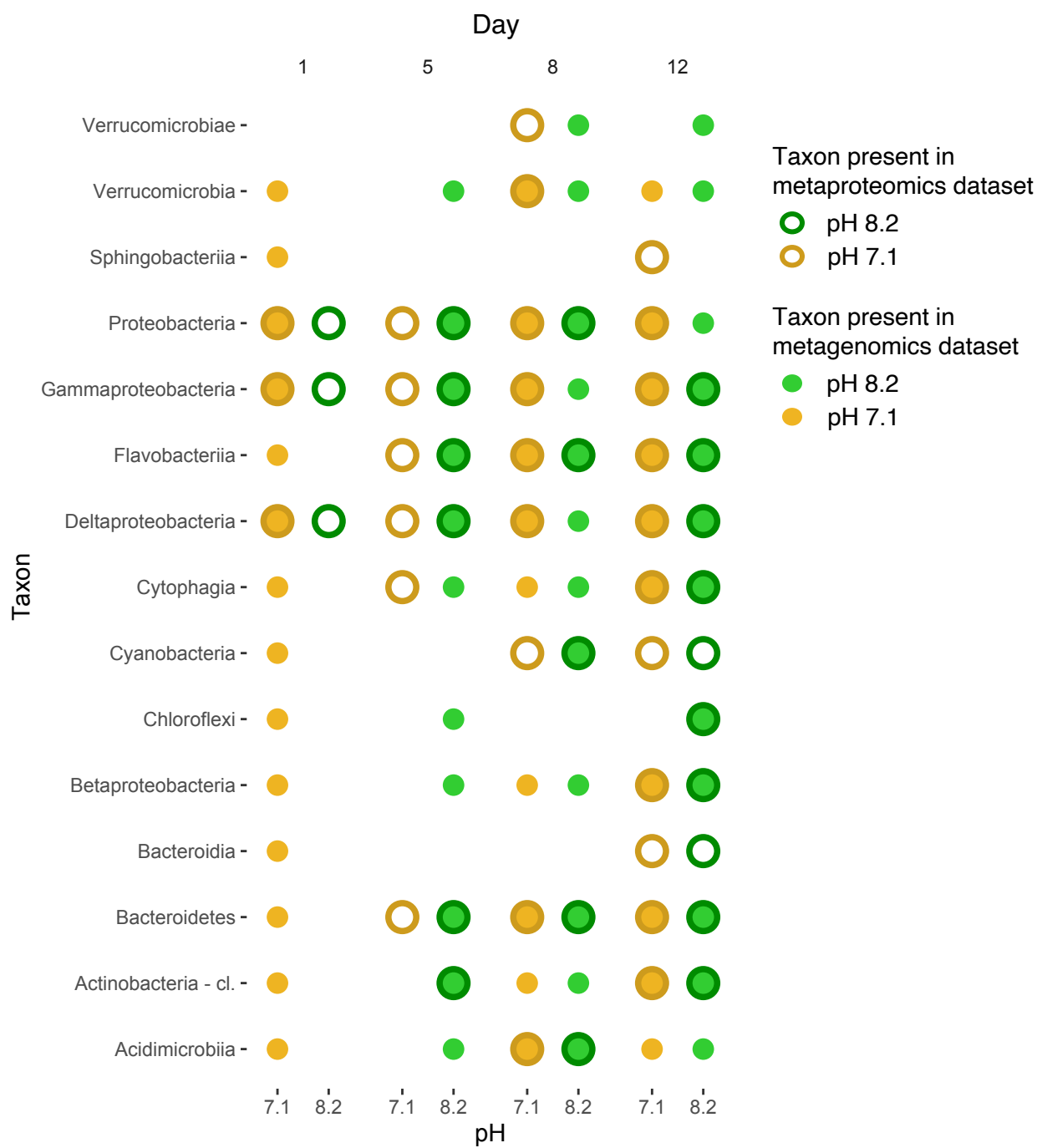

Supplement: Supplementary file 8 — Additional file 8. Representation of tax that are missing from a dataset for a given sampling day or pH. Solid circles indicate that the taxon was present in the metagenomics dataset; an open circle indicates presence in the metaproteomics dataset. [file 40793_2021_376_MOESM8_ESM.pdf]

Ratio of Metaproteomic PSMs in Run

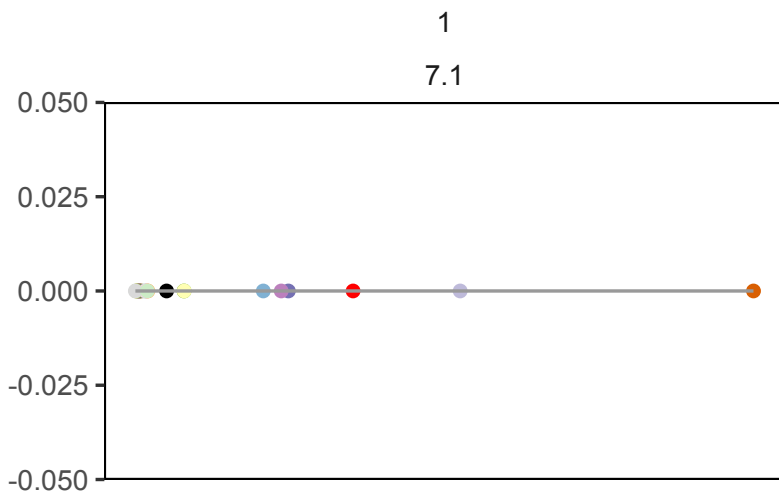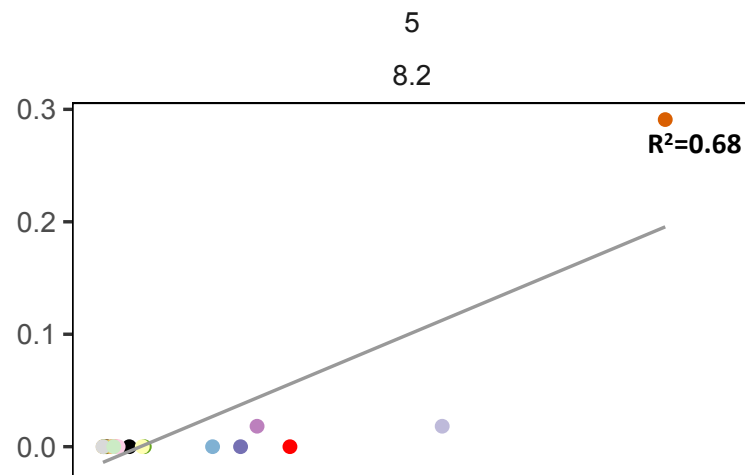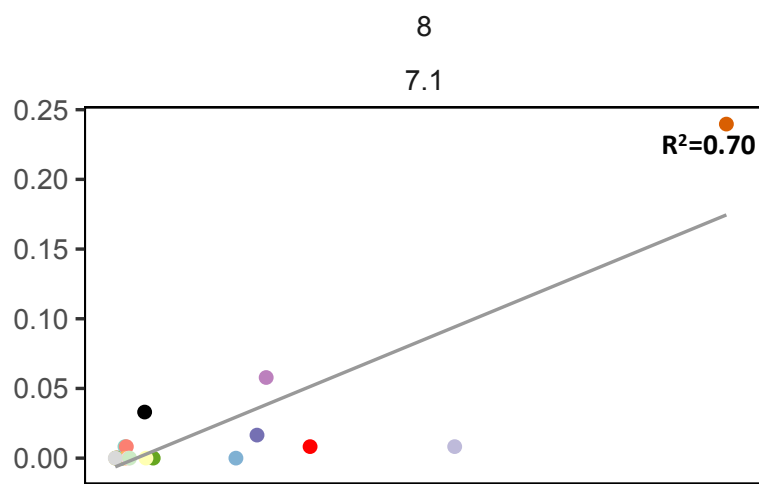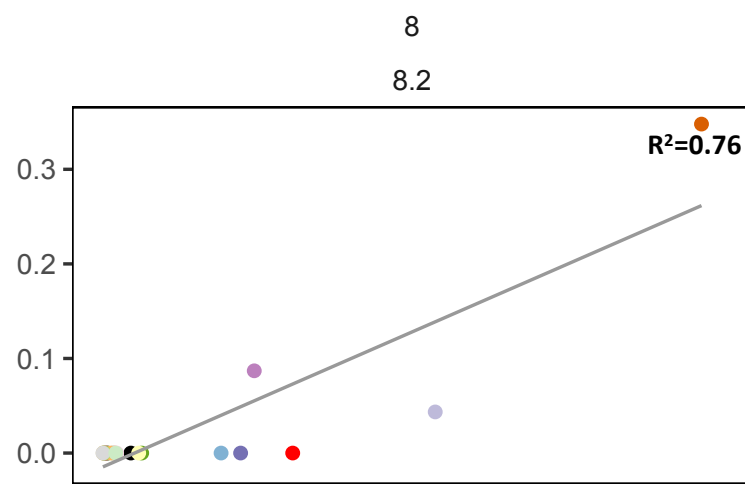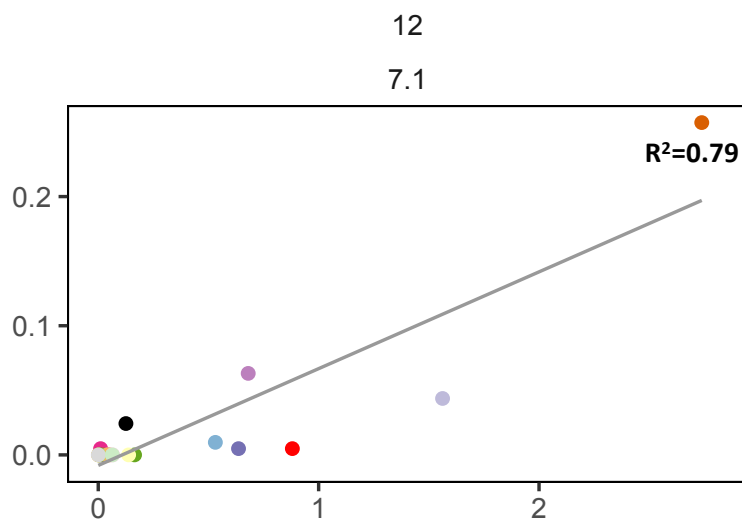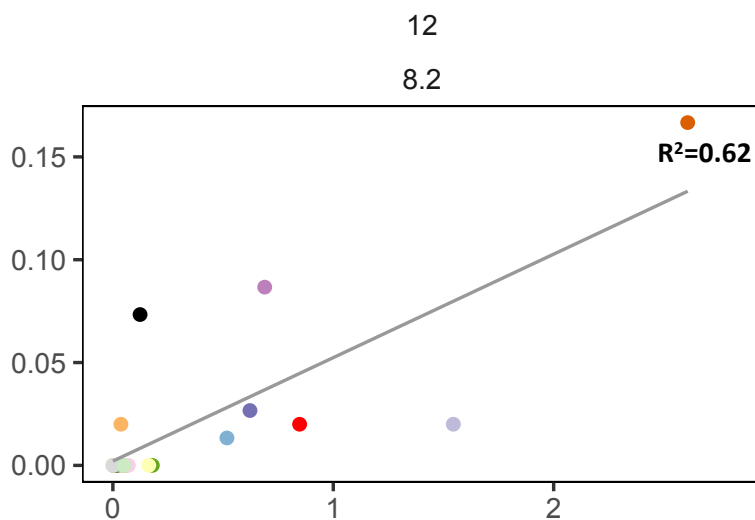

Ratio of Metagenomic Reads in Library

Supplement: Supplementary file 9 — Additional file 9. Plots of the correlation the metagenomic reads and metaproteomic peptide spectral matches for each Gene Ontology term identified for each day and pH in the metagenomics dataset. R2 values are reported on each plot and colors correspond to Fig. 4. [file 40793_2021_376_MOESM9_ESM.pdf]
